# Multilayer Perceptron Neural Network Analysis of Fluoroscopic Working Angle on Transcatheter Aortic Valve Implantation Complications

**DOI:** 10.7759/cureus.59144

**Published:** 2024-04-27

**Authors:** Nathan Asif, Peace Ayoade, Jacob Razzouk, Daniel Bohen, Megan Tooker, Lynne Gladstone, Jason Hoff, Amr Mohsen, Steve Arnold, David G Rabkin

**Affiliations:** 1 Department of Cardiothoracic Surgery, Loma Linda University Medical Center, Loma Linda, USA; 2 Viterbi School of Engineering, University of Southern California, Los Angeles, USA; 3 Department of Internal Medicine, Loma Linda University Medical Center, Loma Linda, USA

**Keywords:** cardiac surgery, aortic valve stenosis, machine learning, surgical complications, trans-catheter valve implantation

## Abstract

Background: We sought to determine whether there is a relationship between the fluoroscopic working angle used to achieve a co-planar view during the deployment of the prosthesis during transcatheter aortic valve implantation (TAVI) and rates of complications, including paravalvular leaks, complete heart block, annular rupture, stroke, valve embolization, discharge to a skilled nursing facility and death within thirty days.

Methods: All patients undergoing TAVI at our institution from 2015 to 2022 were retrospectively analyzed. Images were reviewed to determine the fluoroscopic working angle during deployment, and medical records were used to determine the incidence and type of complication. A multilayer perceptron was employed to evaluate the predictive ability of the fluoroscopic working angle during deployment on complications of one-day and 30-day paravalvular leak, 30-day mortality, the need for a new pacemaker, discharge to a skilled nursing facility, stroke and the requirement for emergency intervention.

Results: Eight hundred and thirty-four patients were included in the study. Fluoroscopic working angle had excellent predictive value for stroke (area under the receiver operating characteristic curve (AUROC) of 0.812), one-day (AUROC 0.850), and 30-day paravalvular leak (AUROC 0.801). However, feature importance and scaled weighting analysis indicated that only a working angle in the left anterior oblique/cranial quadrant was informative for the development of an outcome of interest specific to a working angle quadrant (30-day paravalvular leak).

Conclusion: Fluoroscopic working angle may be a useful way to further refine well-established risk calculi during TAVI.

## Introduction

Since its introduction twenty years ago, transcatheter aortic valve implantation (TAVI) has become increasingly ubiquitous as a therapy for severe, symptomatic aortic stenosis. While results are generally excellent, complications including death, stroke, paravalvular leak, complete heart block and bleeding leading to emergency operative intervention are unavoidable. Over the past decade device modifications in addition to increased operator experience have led to a substantial reduction in most of these complications. Improved patient selection has reduced mortality, newer generation prostheses that have sealing skirts covering the periprosthetic space have reduced the incidence of paravalvular leaks [1], modifications to the positioning of the prostheses have reduced the incidence of complete heart block [2], and a decrease in the profile of the sheaths and deployment catheters have reduced the incidence of major vascular complications [3]. However, accurate risk assessment forms an essential component of patient-centric care, and as TAVI has migrated from high- to low-risk patient populations, risk stratification has become increasingly important.

We hypothesized that given the organization of typical cardiac catheterization laboratories, right anterior oblique and caudal positioning of the fluoroscopic C-arm to achieve a fluoroscopic projection perpendicular to the native valve (the so-called ‘co-planar’ view) results in awkward positioning of the operators during the crucially important deployment of the prosthesis and therefore might also be associated with an increased complication rate. Therefore, although the large prospective, randomized placement of aortic transcatheter valves (PARTNER) trials have documented complication rates following TAVI in various risk populations [4-10], we felt that there could be hidden pockets of complications based on patient-specific anatomy and therefore retrospectively reviewed our TAVI experience to determine whether there is a relationship between cardiac geometry and orientation (using the fluoroscopic working angle as a proxy for the patients’ cardiac axis orientation) on the development of complications after TAVI.

## Materials and methods

**Figure 1:**
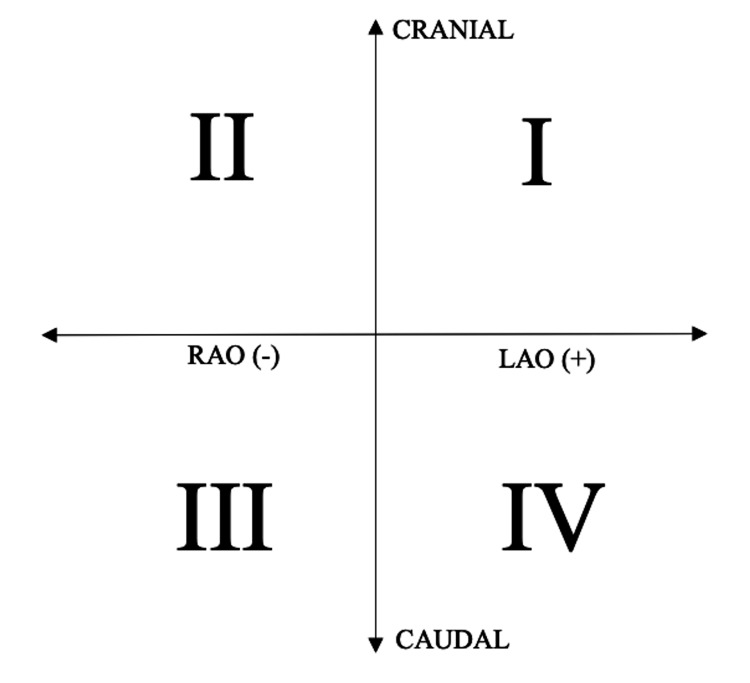
Coordinate system used for fluoroscopic working angle and labeling of quadrants. RAO: right anterior oblique, LAO: left anterior oblique. This figure is the original work of the authors.

We reviewed the electronic medical records of all patients undergoing TAVI at our institution between October 2013 and May 2022. The patients’ vital statistics, comorbidities, baseline left ventricular ejection fraction, native aortic valve morphology, and STS score were recorded in an anonymized database. Fluoroscopic images were reviewed to determine the working angle of the fluoroscopy C-arm during TAVI deployment (right vs. left anterior oblique (RAO/LAO) and cranial vs. caudal (CRAN/CAUD)), which were then converted to a Cartesian coordinate system (Figure 1).

In addition to the coordinate points along the anterior oblique and craniocaudal axes, TAVI angle characteristics were further characterized as follows: quadrant (Q) location in the Cartesian coordinate system (Q1, Q2, Q3, or Q4), and distance from the origin of the Cartesian system, defined as point (0,0) along the anterior oblique and craniocaudal axes. 

Post-procedure echocardiogram reports were reviewed to determine the presence or absence of a 1-day and/or 30-day paravalvular leak and, if present, their severity. Medical records were used to determine the incidence of other TAVI complications, including the need for a new permanent pacemaker, 30-day mortality, stroke, discharge to a skilled nursing facility (SNF), and the requirement for emergency surgical intervention; if present, they were recorded in an anonymized database.

Our study design was submitted to the Institutional Review Board at Loma Linda University Medical Center, which determined that the study did not meet the definition of human subject research because it did not involve identifiable information, no data or specimens were collected and there was no direct intervention or interaction. Therefore, the Institutional Review Board decided our study did not require review or approval.

Machine learning

We utilized machine learning modeling to predict each of the post-TAVI complications analyzed in this study using solely the characteristics of the TAVI working angle, irrespective of patient medical, demographic, or anthropometric history. For each model, a stratified 70/30 train test split was used with the initial architecture search performed using Hyperopt, a Python library built for automatic model selection and hyperparameter optimization [11]. Hyperopt uses meta-learning based on Bayesian optimization methods to provide solutions in a combined algorithm selection and hyperparameter optimization approach. Hyperopt evaluated the performance of linear regression, random forest, extra trees, stochastic gradient descent, ada boost, k-nearest-neighbors, Gaussian Naive Bayes, Bernoulli Naive Bayes, and multilayer perceptron (MLP) algorithms for 10,000 iterations each. Following this, the best-performing model for prediction of all outcomes was determined to be MLP. Having one model type consistently perform superiorly across all outcomes, as opposed to different models performing better for various outcomes, makes sense in terms of performance as it signifies that the MLP model type performed well on the underlying distribution of the features rather than performing well based on the distribution of outcomes.

MLP is a type of artificial neural network (ANN) that consists of multiple layers of interconnected artificial neurons, or nodes. It is a feedforward neural network, meaning that information flows through it in one direction, from the input layer to the output layer, without any loops or cycles. The MLP architecture consists of an input layer, one or more hidden layers, and an output layer. Each layer, except the input layer, is composed of multiple nodes, also known as perceptrons or artificial neurons. The nodes in each layer are fully connected to the nodes in the subsequent layer, meaning that each node receives input from all the nodes in the previous layer. Each node in an MLP performs a weighted sum of its inputs, adds a bias term, and then applies an activation function to produce an output. The activation function introduces non-linearity into the network, enabling it to learn complex patterns and relationships in the data. Common activation functions used in MLPs include sigmoid, hyperbolic tangent (tanh), and rectified linear unit (ReLU). During the training process, the weights and biases of the MLP are adjusted based on a supervised learning algorithm such as backpropagation. Backpropagation computes the gradients of the network's output with respect to its weights and biases and updates them in a way that minimizes a defined loss function, typically using gradient descent optimization. MLPs are versatile and can be applied to a wide range of tasks, including classification, regression, and pattern recognition.

The small size of the dataset allowed for rapid model training and iteration, and we selected a combination of the area under the receiver operating characteristic curve (AUROC) and balanced accuracy as our loss function to help assist in avoiding model overfitting. Due to the small size of the dataset and the fact that it was heavily imbalanced, we employed an adaptive synthetic sampling approach on each training split. This oversamples the minority class by creating similar synthetic samples and adding them to the training set. This allows the model to avoid overfitting and being heavily biased toward the majority class while retaining the integrity of the model as none of the synthetic data is used in the test set. In every case, this resulted in oversampling the complication for training the model, before evaluating its performance on the unbiased test set. 

To assess ML model performance, the following standard metrics were evaluated: AUROC, accuracy, balanced accuracy, precision, recall, and F1 score [12]. Regarding the use of accuracy and balanced accuracy, the imbalanced nature of the dataset indicated accuracy was not as reliable of a measure as balanced accuracy, which still falls short of AUROC [13]. Accuracy measures only the percent correctly classified. In the case of a paravalvular leak within 30 days, for example, 98.8% accuracy could be achieved by simply always predicting no leak, as only 1.2% of cases demonstrated a leak. In contrast, AUROC is a measure of how well a model can distinguish between classes, with a measure of 0.5 corresponding to random chance. We considered AUROC above 0.800 to be highly predictive and classified it as “excellent.” AUROC is robust against class imbalances, as it takes into account true and false positive rates. 

The importance of each variable can be determined based on permutation feature importance, defined as the decrease in model score when a given variable is removed from the algorithm [14]. Therefore, feature importance and scaled weights for each TAVI angle characteristic were also computed. Weights were scaled from −1 to 1, with a negative value signifying the given TAVI angle characteristic is associated with a decreased probability of a given outcome or complication occurring. Matplotlib was used for graphic visualization of the AUROC produced by the modeling [15]. 

## Results

**Figure 2:**
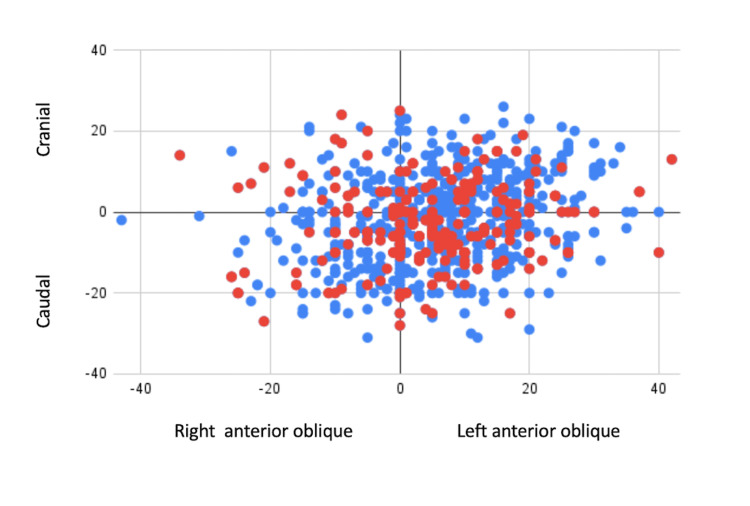
Patients with and without any complications by fluoroscopic working angle quadrant. Blue dots represent patients without any complications, and red dots represent patients with any complications.

Of the 834 patients included in this study, 33.0% had co-planar fluoroscopic views in the LAO/cranial quadrant, 35.0% in the LAO/caudal quadrant, 10.8% in the RAO/cranial quadrant, and 20.8% in the RAO/caudal quadrant. Fourteen demonstrated paravalvular leak within one day of TAVI, 10 demonstrated a paravalvular leak at 30 days, there were 15 instances of stroke, 41 of hematoma, 20 of mortality within 30 days, 77 who required a pacemaker, and 87 who were transferred to a SNF following TAVR (Figure 2). 

The performances of each machine learning model for prediction of hematoma, discharge to SNF, stroke, pacemaker, one-day and 30-day PL, and 30-day mortality are reported in Table 1.

**Table 1:** Model performance using TAVI working angle characteristics for prediction of complications following TAVI. TAVI: transcatheter aortic valve implantation, AUROC: area under receiver operator characteristics.

Complication	AUROC	Accuracy	Balanced accuracy	Precision	Recall	F1 score
Hematoma	0.647	0.932	0.569	0.222	0.167	0.190
Discharge to the skilled nursing facility	0.607	0.607	0.607	0.151	0.615	0.242
Stroke	0.812	0.824	0.812	0.085	0.800	0.154
One-day paravalvular leak	0.850	0.696	0.723	0.038	0.750	0.073
30-day paravalvular leak	0.801	0.932	0.801	0.111	0.667	0.190
30-day mortality	0.760	0.532	0.760	0.049	1.000	0.093
Pacemaker	0.625	0.358	0.626	0.121	0.957	0.217

The associated feature importances and scaled weighting of the TAVR working angle characteristics are displayed in Tables 2, 3 for each predicated complication, respectively.

**Table 2:** Feature importance of TAVI working angle characteristics for the prediction of complications after TAVI. RAO: right anterior oblique, LAO: left anterior oblique, CAUD: caudal, CRAN: cranial, TAVI: transcatheter aortic valve implantation.

Characteristics	Hematoma	Discharge to the skilled nursing facility	Stroke	One-day paravalvular leak	30-day paravalvular leak	30-day mortality	Pacemaker
Location in Q1	0.130	0.143	0.167	0.170	0.458	0.650	0.198
Location in Q2	0.192	0.143	0.139	0.182	0.083	0.003	0.106
Location in Q3	0.142	0.143	0.139	0.241	0.098	0.014	0.144
Location in Q4	0.136	0.145	0.139	0.104	0.116	0.035	0.195
Distance from origin	0.133	0.142	0.139	0.101	0.073	0.117	0.133
RAO (−)/LAO (+)	0.132	0.142	0.139	0.101	0.084	0.080	0.107
CAUD (−)/CRAN (+)	0.134	0.142	0.139	0.101	0.088	0.103	0.118

**Table 3:** Scaled weighting of TAVI working angle characteristics for prediction of complications after TAVI. RAO: right anterior oblique, LAO: left anterior oblique, CAUD: caudal, CRAN: cranial, TAVI: transcatheter aortic valve implantation.

Characteristics	Hematoma	Discharge to the skilled nursing facility	Stroke	One-day paravalvular leak	30-day paravalvular leak	30-day mortality	Pacemaker
Location in Q1	1.000	0.305	1.000	1.000	1.000	−1.000	0.679
Location in Q2	0.821	0.521	0.666	−1.000	−0.816	0.079	−0.474
Location in Q3	0.133	0.522	0.666	0.796	−0.885	−0.223	1.000
Location in Q4	0.390	0.447	0.666	−0.463	−0.782	−0.690	−0.973
Distance from origin	−1.000	1.000	0.259	−0.271	−0.918	0.980	−0.902
RAO (−)/LAO (+)	−0.498	−1.000	−1.000	−0.473	−0.786	1.000	−0.881
CAUD/CRAN	−0.349	0.665	0.679	−0.322	−1.000	0.107	−1.000

**Figure 3:**
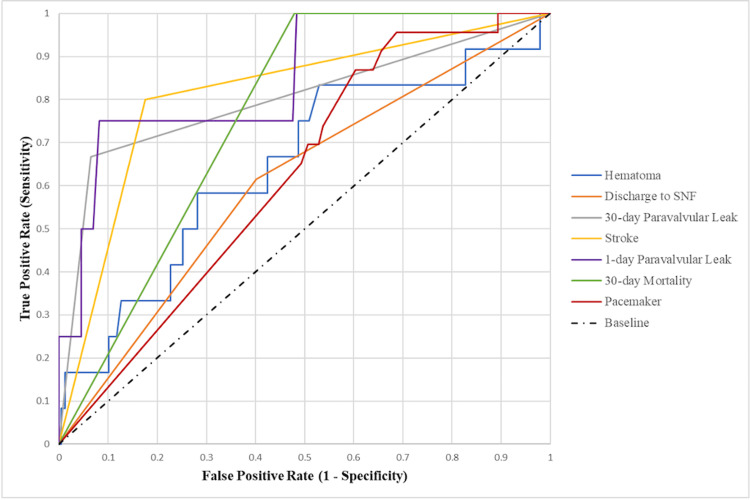
Area under receiver operator characteristic curves for prediction of complications after TAVI. SNF: skilled nursing facility, TAVI: transcatheter aortic valve implantation.

The associated AUC curves for each predicated complication are displayed in Figure 3.

MLP modeling performance was highly predictive for stroke (AUROC of 0.812), one-day paravalvular leak (AUROC of 0.850), and 30-day paravalvular leak (AUROC of 0.801). Feature importance demonstrated that only 30-day paravalvular leak had a marked preponderance for an imputed characteristic (0.458 for location in Q1 vs. 0.116 for next highest characteristic location in Q4), while feature importance for stroke and paravalvular leak showed markedly less of a quadrant preponderance (stroke: 0.167 location in Q1 vs. 0.139 for all other characteristics and one-day paravalvular leak 0.241 location in Q3 vs. next highest characteristic 0.182 location in Q2). 

## Discussion

To our knowledge, the relationship between the fluoroscopic working angle employed to achieve a co-axial view of the aortic annular anatomy and complication rates has not been reported. Our study suggests there may be associations between the fluoroscopic working angle and the development of certain types of complications. Specifically, we found that annular orientation requiring a fluoroscopic working angle in the left anterior oblique-cranial projection was most informative for the prediction of post-procedure stroke and paravalvular leak at 30 days, while the right anterior oblique-caudal projection was most informative for prediction of paravalvular leak at one day. The permutation feature importance analysis suggests that while there was an association between the working angle and post-TAVI stroke and paravalvular leak at one day, the weighting was relatively evenly distributed, suggesting a multitude of factors were equally influential in the algorithm’s decision-making process. The feature analysis of the association between working angle and paravalvular leak at 30 days, however, was considerably more weighted on quadrant I (0.458), suggesting a working angle located in quadrant I was most informative for the algorithm’s decision-making process. Other complications, including the development of a hematoma, discharge to a skilled nursing facility, the need for permanent pacemaker implantation, and 30-day mortality, did not meet the definition for having a robust classification with respect to the fluoroscopic working angle (AUROC > 0.800). 

It is understandable that the development of a groin hematoma was unrelated to the fluoroscopic working angle, as hematoma formation is more likely related to the quality and size of the femoral vessels, the technique of insertion, the method of hemostasis, and the patient’s coagulation profile. Discharge to a skilled nursing facility, while perhaps indirectly related to complications, is confounded by other aspects of a patient’s profile, such as their social support, home living arrangement, type of insurance, and financial resources. The need for a new-onset permanent pacemaker, while related to prosthesis placement, has been shown to be lower with a 90/10 aortic/ventricle distribution compared to an 80/20 [2], and therefore accurate working angle deployment to implant the prosthesis precisely is critical. It is also likely confounded by the more important risk factor of the pre-existing right bundle branch block, which is unrelated to annular orientation. Other risk factors for heart block during TAVI include the utilization of large valves with resultant large valves indexed to patients' body surface area and the use of self-expanding valves (which were rarely used in our study) are also unrelated to the fluoroscopic working angle [16]. Mortality at 30 days is likely significantly confounded by patients’ comorbidities at the time of the procedure.

Our hypothesis that a working angle in the right anterior oblique-caudal quadrant would be associated with higher complication rates due to the typical setup of cardiac catheterization laboratories resulting in the fluoroscopic equipment compromising the operators’ access to the device for manipulation and balloon inflation during deployment did not bear out. Explanations for why the left anterior oblique-cranial projection was most informative for the prediction of a thirty-day paravalvular leak are speculative at best. While human anatomy varies considerably, normally the left ventricle is positioned posteriorly, and the aorta typically emerges from the left ventricle directed anteriorly. Therefore, a co-axial projection would typically require caudal positioning of the fluoroscopy equipment. Similarly, to orient the right coronary sinus (which comes off the aorta anteriorly and slightly to the right) between the non- and left-coronary sinuses a slight rightwards orientation of the fluoroscopy equipment is typically required. While certainly variations occur and a left anterior oblique or a cranial projection is not that unusual (in fact, the LAO/cranial quadrant was the second most common, accounting for 33% of the patients in our study), it is possible that the combination, a predicted left anterior oblique-cranial projection, may be more likely to inaccurately capture a true co-axial projection and therefore increase the rate of paravalvular leak improper positioning. 

Limitations of our study include its single-center, retrospective nature and the relatively small dataset and low number of each type of complication, which we tried to compensate for with our statistical analysis as described in the Methods section. The study also included a seven-year period of time during which, due to changes in the risk burden to qualify for TAVI, there was a shift towards a higher proportion of younger, healthier patients towards the end of the study interval. In addition, as noted earlier, there were improved device modifications and increased operator experience over this time.

A final limitation of our study is its inability to fully account for the “black box” nature of the algorithm’s decision-making process-the technical term for models that produce predictions without providing a clear understanding of how their conclusions are reached. While we attempted to uncover the model’s decision-making process via analysis of its permutation feature importance and scaled weighting metrics, nevertheless, there remains an element of ambiguity pertaining to how the model was able to make its predictions. Regardless, the high performance of the modeling’s predictions indicates that the fluoroscopic working angle is a relevant factor with respect to the development of complications. Therefore, while this is a pilot study, our findings suggest the fluoroscopic working angle can influence the development of complications post-TAVI and, therefore, warrant further investigation and optimization. This is especially true given that the modeling was able to predict complications with high performance without the use of other informative data elements such as patient medical, demographic, or anthropometric characteristics. 

## Conclusions

Fluoroscopic working angle during trans-catheter aortic valve implantation may be a useful adjunct to currently available risk calculi regarding the presence of paravalvular leak at 30 days. Our work justifies a larger, multi-center, prospective study. Should our preliminary results be confirmed in large trials, they may influence the decision to employ transcatheter versus surgical aortic valve procedures, particularly in patients at low risk for surgical aortic valve replacement.
